# Psoas and Thigh Abscesses As Rare Complications of Cesarean Delivery: A Case Report

**DOI:** 10.7759/cureus.115018

**Published:** 2026-08-23

**Authors:** Benabdeslam Rim, Anibri Mouna, Safae Benjelloun, Khalid Fathi

**Affiliations:** 1 Obstetrics and Gynecology, Oncology and High-Risk Pregnancy, Souissi Maternity Hospital, Rabat, MAR; 2 Obstetrics and Gynaecology, Faculty of Medicine and Pharmacy of Rabat, Rabat, MAR

**Keywords:** case report, cesarean section, iliopsoas abscess, postpartum infection, thigh abscess

## Abstract

Iliopsoas abscess is an uncommon but potentially severe condition in obstetric practice, posing significant diagnostic challenges due to its deep anatomical location and nonspecific clinical presentation. Its occurrence in the postpartum period, particularly following cesarean delivery, remains exceptionally rare.

We report the case of a 32-year-old woman who developed an extensive left iliopsoas abscess with lumbar and thigh extension one week after cesarean section performed for prolonged rupture of membranes. She presented with high-grade fever, severe low back pain, and a tender lumbar swelling complicated by spontaneous fistulization to the thigh. Contrast-enhanced CT demonstrated a large lumbar abscess communicating with a retroperitoneal collection involving the iliopsoas muscle. The patient underwent prompt surgical drainage, yielding approximately 1 L of purulent material, with microbiological culture isolating *Staphylococcus aureus*. Combined surgical and broad-spectrum antibiotic therapy resulted in a favorable clinical outcome with complete resolution at follow-up.

This case underscores the importance of maintaining a high index of suspicion for iliopsoas abscess in postpartum patients presenting with persistent fever and lumbosacral or thigh pain after cesarean delivery. Early imaging, timely intervention, and multidisciplinary management are critical to reducing morbidity and ensuring optimal outcomes.

## Introduction

Iliopsoas abscess is defined as a collection of purulent material within the iliopsoas compartment. It is an uncommon but potentially serious condition, particularly rare in obstetric practice [1,2]. Iliopsoas abscesses are traditionally classified as primary or secondary. Primary abscesses result predominantly from hematogenous dissemination, with *Staphylococcus aureus* being the most frequently implicated pathogen, whereas secondary abscesses arise from contiguous spread of infection from adjacent structures or may occur in association with surgical or invasive procedures [1,2].

The clinical presentation is often nonspecific because of the deep retroperitoneal location of the iliopsoas muscle. Fever, back or flank pain, hip or thigh pain, and limitation of hip movement may occur, whereas the classical triad of fever, back pain, and limp is present in only a minority of patients [3]. Consequently, diagnosis may be delayed, particularly in postpartum patients in whom fever and pain may initially be attributed to more common puerperal conditions.

Postpartum iliopsoas abscess has been only rarely reported, including cases occurring during pregnancy, following curettage or spontaneous abortion, and during the postpartum period [4,5]. Only isolated cases following cesarean delivery have been described [6,7]. Geirsson also highlighted the rarity of psoas abscess in association with pregnancy and cesarean delivery [8]. More recent reports have continued to document iliopsoas abscess as an unusual cause of postpartum infection and sepsis [9,10]. Consequently, the clinical spectrum, routes of infectious spread, and optimal diagnostic approach to postcesarean iliopsoas abscess remain poorly characterized.

The classical clinical presentation of iliopsoas abscess remains uncommon, contributing to diagnostic delay [11]. Pain may radiate to the hip or thigh because of the anatomical relationship of the iliopsoas muscle with the lumbar plexus [12]. Cross-sectional imaging, particularly contrast-enhanced CT, plays a central role in establishing the diagnosis, determining the extent of infection, identifying potential fistulous tracts, and guiding drainage [3,13].

Several predisposing factors for iliopsoas abscess have been described, including diabetes mellitus, immunosuppression, local infection, and recent surgery. In the obstetric setting, cesarean delivery may provide a potential setting for deep infection through postoperative or contiguous spread from a pelvic infectious focus. In addition, prolonged rupture of membranes and postpartum endometritis are recognized contributors to postpartum infectious morbidity and may potentially facilitate the development or extension of deep pelvic and retroperitoneal infection. However, the precise pathophysiological mechanisms underlying postcesarean iliopsoas abscess remain incompletely understood because of the extreme rarity of this complication.

We report an unusual case of an extensive left iliopsoas abscess occurring one week after cesarean delivery performed in the context of prolonged rupture of membranes, with concomitant postpartum endometritis and extension into the lumbar subcutaneous tissues and thigh with spontaneous fistulization. The extensive anatomical spread and fistulous communication observed in this case illustrate an unusual presentation of this rare postcesarean complication. This report highlights the importance of considering iliopsoas abscess in postpartum patients presenting with persistent fever associated with lumbar, flank, hip, or thigh symptoms and emphasizes the role of early cross-sectional imaging and multidisciplinary management.

## Case presentation

A 32-year-old woman (G3P3), weighing 60 kg, underwent cesarean delivery following prolonged rupture of membranes lasting more than 24 hours. Her medical history was notable only for gestational diabetes managed with dietary measures, and she had no known immunodeficiency.

One week after cesarean delivery, she was readmitted with severe low back pain, chills, and fluctuating fever reaching 39°C. The exact timing of symptom onset during the postpartum period was not documented in the available medical record. On admission, physical examination revealed a tender, erythematous, warm swelling measuring approximately 10 × 9 cm in the left lumbar region. The Pfannenstiel incision was clean, and abdominal examination was otherwise unremarkable. Gynecological examination revealed foul-smelling yellow vaginal discharge and uterine tenderness on mobilization. A superficial thigh abscess with spontaneous fistulization was also present.

Laboratory investigations on admission showed a hemoglobin level of 9.6 g/dL, a white blood cell count of 9.6 × 10⁹/L, and a markedly elevated C-reactive protein level of 247 mg/L (Table 1). Thus, despite a white blood cell count within the normal reference range, the markedly elevated C-reactive protein level supported a significant inflammatory process.

**Table 1:** Laboratory findings at admission, with corresponding reference ranges and interpretation

Parameter	Admission	Reference range	Interpretation
Hemoglobin	9.6 g/dL	12–16 g/dL	Decreased
White blood cell count	9.6 ×10⁹/L	4–10 ×10⁹/L	Normal
C-reactive protein	247 mg/L	<5 mg/L	Increased

Given the postpartum fever, foul-smelling vaginal discharge, and uterine tenderness, postpartum endometritis was initially suspected. However, the association of severe lumbar pain, a large inflammatory lumbar swelling, and thigh involvement raised concern for a deeper infectious process extending beyond an isolated uterine infection.

Pelvic ultrasound demonstrated findings suggestive of postpartum endometritis and identified a rounded heterogeneous mass adjacent to the left flank (Figure 1A). Contrast-enhanced CT was subsequently performed and demonstrated a large left lumbar subcutaneous abscess measuring 87 × 118 × 140 mm, communicating through a fistulous tract with a deep retroperitoneal collection involving the left iliopsoas muscle (Figure 1B-1D). These findings established the diagnosis of an extensive left iliopsoas abscess with lumbar and thigh extension.

**Figure 1:**
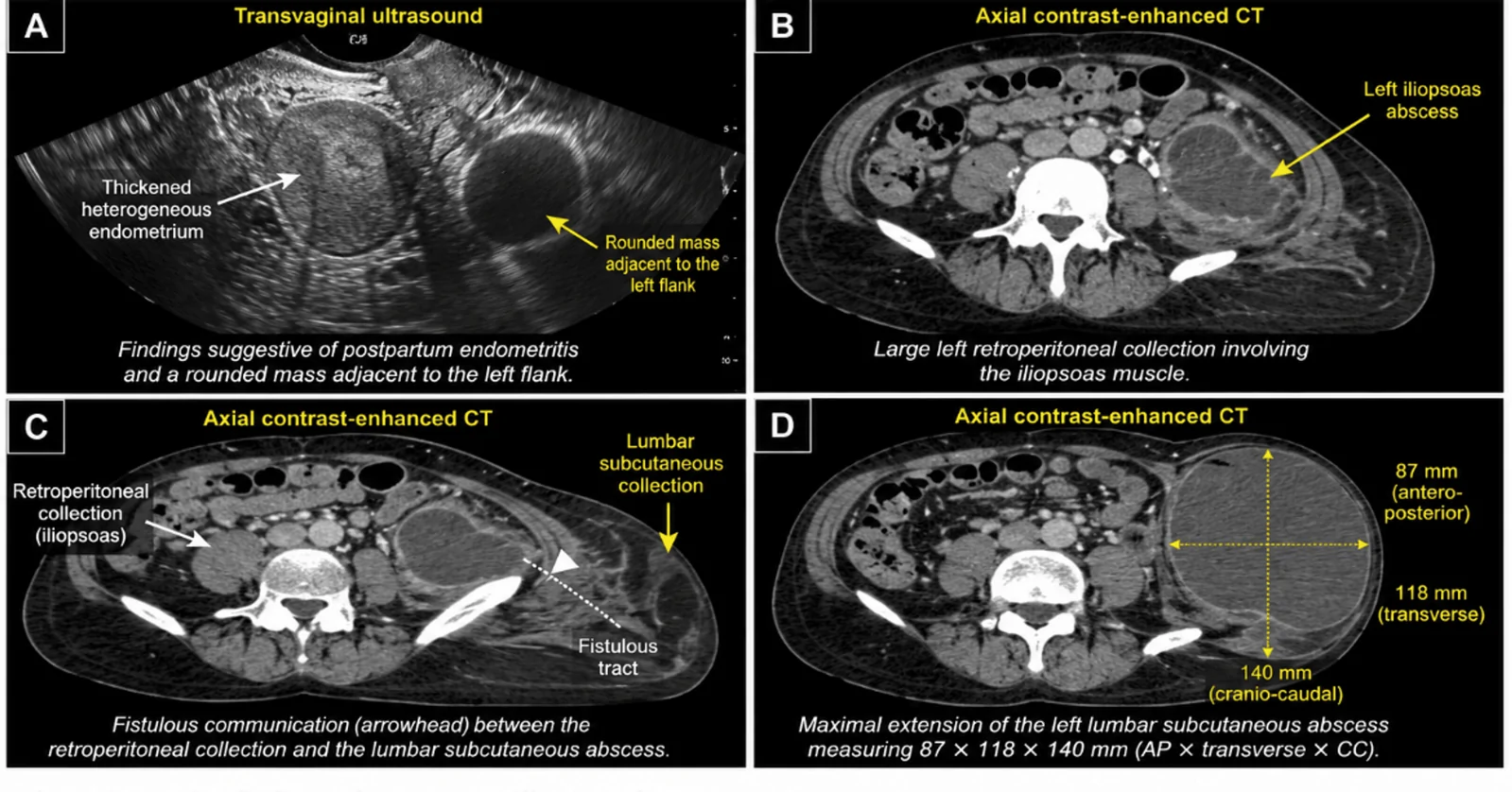
Multimodality imaging of postpartum iliopsoas abscess with lumbar extension (A) Transvaginal ultrasonography showing findings consistent with postpartum endometritis and a rounded heterogeneous collection adjacent to the left flank; (B) Contrast-enhanced axial CT demonstrating a deep left retroperitoneal collection involving the iliopsoas muscle, consistent with an iliopsoas abscess; (C) Contrast-enhanced axial CT demonstrating the fistulous communication between the deep retroperitoneal iliopsoas collection and the superficial lumbar subcutaneous abscess; (D) Contrast-enhanced axial CT demonstrating the extensive left lumbar subcutaneous component of the abscess, measuring approximately 87 × 118 × 140 mm

Empirical broad-spectrum antimicrobial therapy with ceftriaxone, gentamicin, and metronidazole was initiated. Following multidisciplinary consultation, surgical drainage was performed, with evacuation of approximately 1 L of purulent material (Figures 2-4). Microbiological culture of the drained material grew *Staphylococcus aureus*. Detailed antimicrobial susceptibility testing results and subsequent antibiotic adjustments were not available in the medical record.

**Figure 2:**
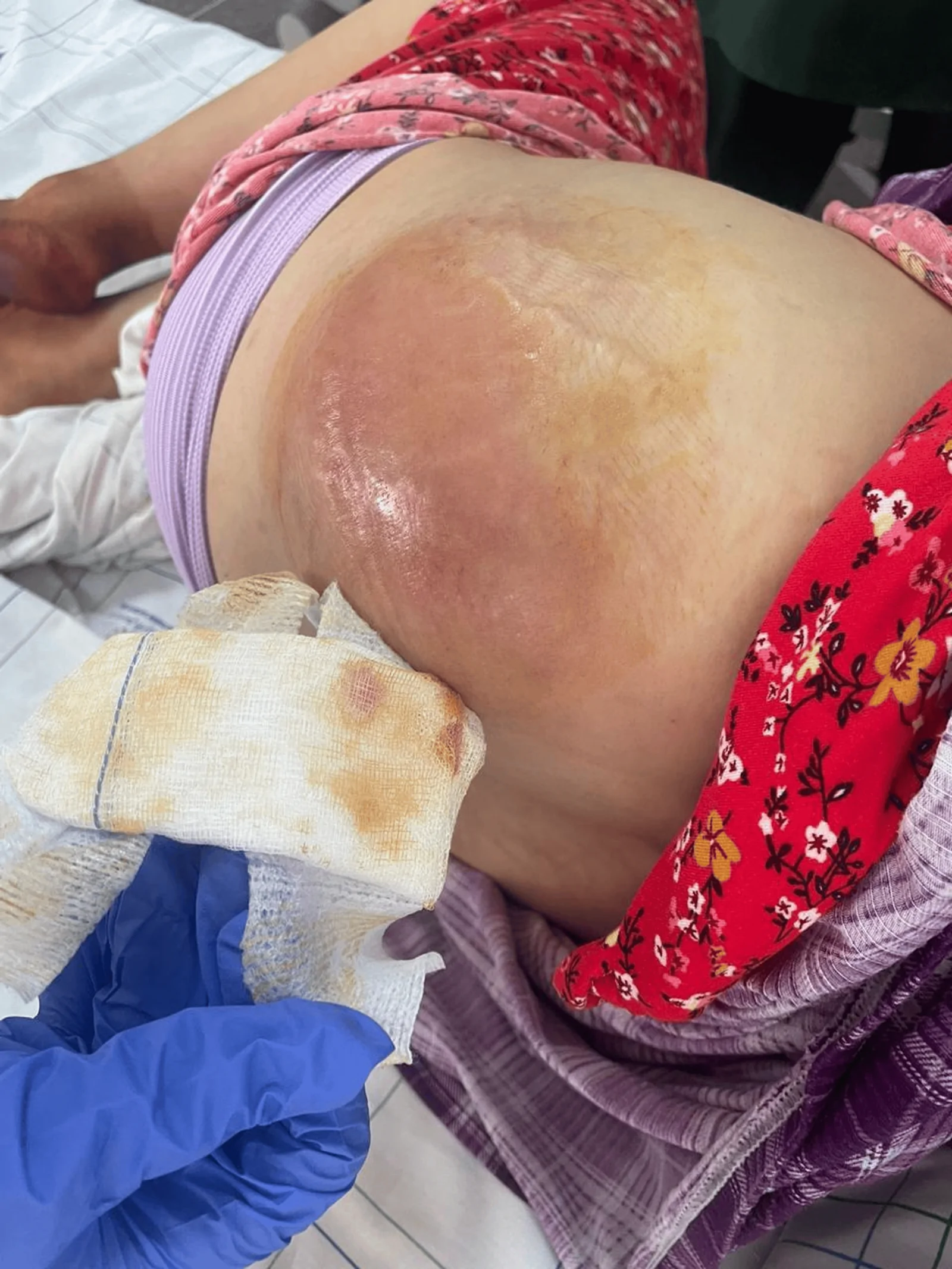
Clinical appearance of the left lumbar region before surgical drainage Preoperative clinical photograph demonstrating a large inflammatory swelling of the left lumbar region, with marked erythema, skin tension, and localized inflammatory changes corresponding to the superficial extension of the deep infectious process

**Figure 3:**
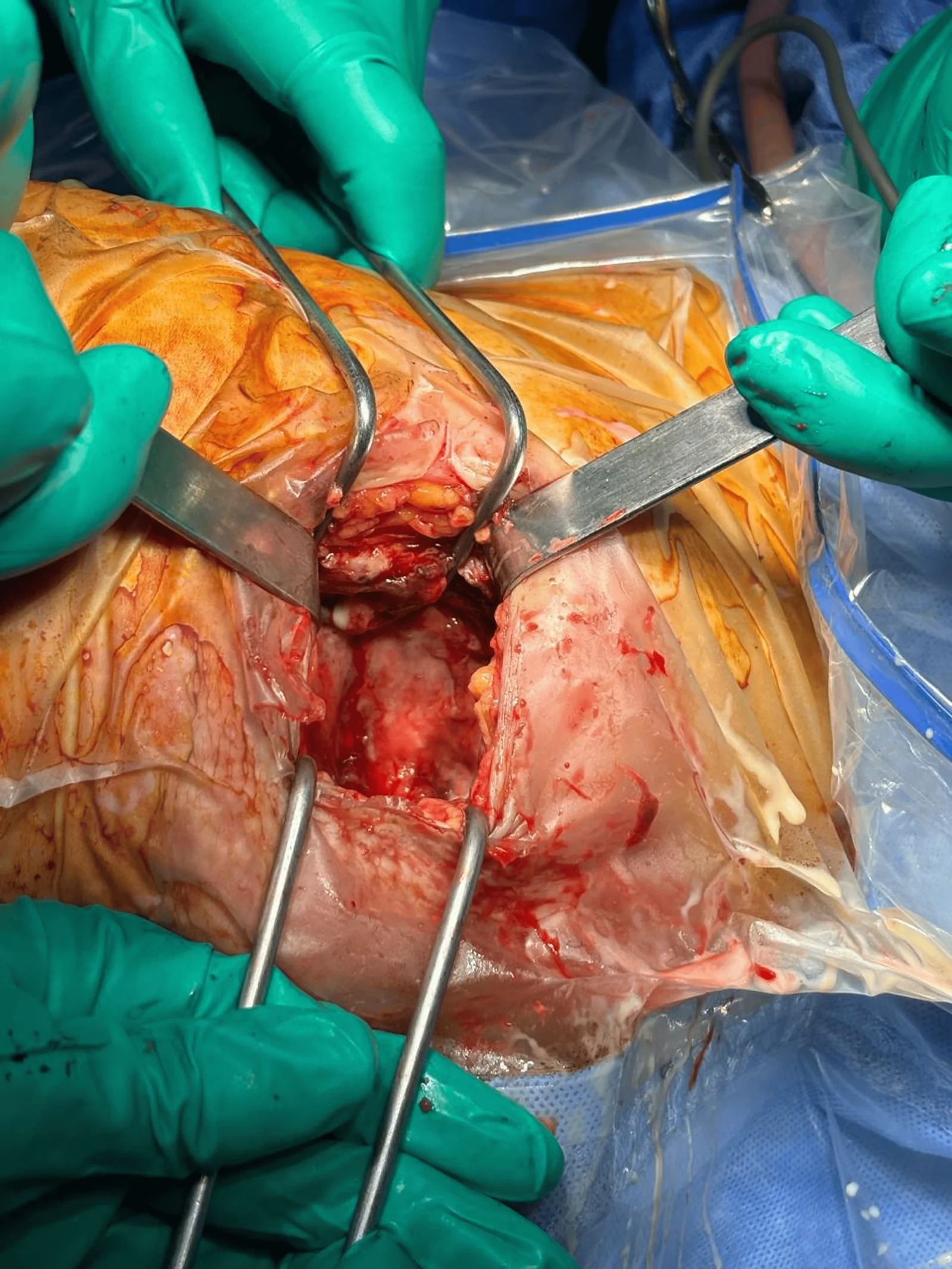
Intraoperative appearance during surgical drainage of the left iliopsoas-associated abscess Intraoperative photograph obtained during posterior surgical drainage showing the opened abscess cavity after incision and evacuation of extensive purulent material. The cavity was subsequently irrigated and a drain was placed.

**Figure 4:**
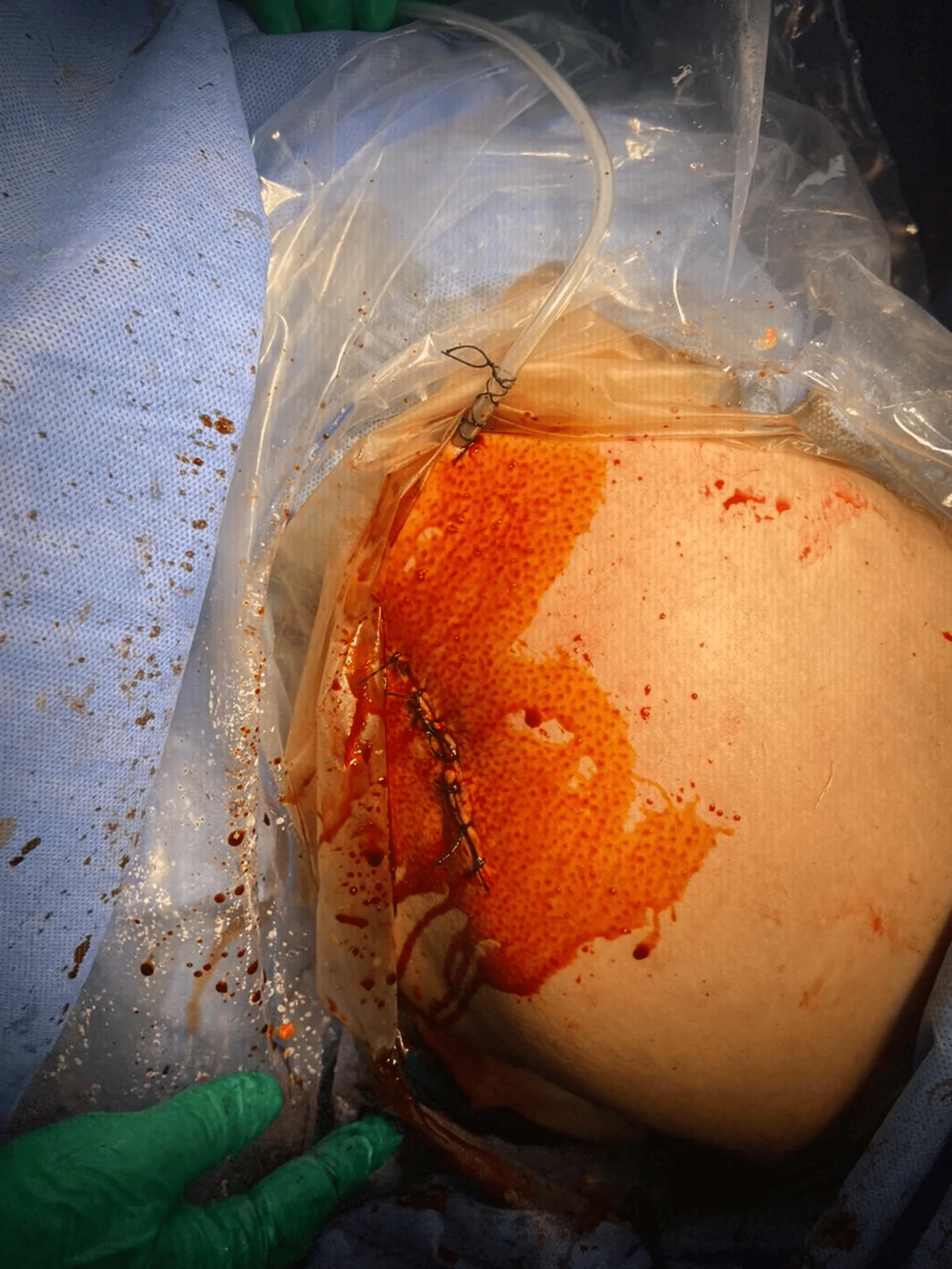
Immediate postoperative appearance following surgical drainage Clinical photograph of the posterior surgical site after evacuation and debridement of the abscess. The wound was closed after adequate drainage of the purulent collection.

The postoperative course was uneventful, and the patient was discharged one week after surgical drainage. At the one-month follow-up, she was asymptomatic, with complete clinical resolution of the infection. The chronological sequence of the clinical course, from cesarean delivery and symptom onset through readmission, diagnostic evaluation, antimicrobial therapy, surgical drainage, and recovery, is summarized in Table 2.

**Table 2:** Clinical timeline from cesarean delivery to recovery Clinical timeline summarizing the sequence from cesarean delivery and symptom onset through readmission, diagnostic evaluation, antimicrobial therapy, surgical drainage, and clinical recovery.

Time point	Clinical findings, investigations, and management
Day 0 – Cesarean delivery	Cesarean delivery was performed following prolonged rupture of membranes lasting >24 hours.
Postpartum period – Symptom onset	Fever, chills, and severe low back pain developed after cesarean delivery. The exact day of symptom onset was not documented in the available medical record.
Postpartum Day 7 – Readmission	The patient was readmitted with severe low back pain, chills, and fluctuating fever reaching 39°C. Examination revealed a tender, erythematous, warm left lumbar swelling measuring approximately 10 × 9 cm, foul-smelling yellow vaginal discharge, uterine tenderness, and a superficial thigh abscess with spontaneous fistulization. The Pfannenstiel incision was clean.
Postpartum Day 7 – Laboratory assessment	Hemoglobin was 9.6 g/dL, white blood cell count was 9.6 × 10⁹/L, and C-reactive protein was markedly elevated at 247 mg/L.
Diagnostic assessment	Postpartum endometritis was initially suspected. The presence of severe lumbar pain, a large inflammatory lumbar swelling, and thigh involvement raised concern for a deeper infectious process.
Imaging assessment	Pelvic ultrasound showed findings suggestive of postpartum endometritis and a heterogeneous mass adjacent to the left flank. Contrast-enhanced CT demonstrated an 87 × 118 × 140 mm left lumbar subcutaneous abscess communicating through a fistulous tract with a deep retroperitoneal collection involving the left iliopsoas muscle, establishing the diagnosis of an extensive iliopsoas abscess with lumbar and thigh extension.
Initial antimicrobial treatment	Empirical broad-spectrum antimicrobial therapy with ceftriaxone, gentamicin, and metronidazole was initiated.
Following diagnostic assessment – Definitive treatment	After multidisciplinary consultation, surgical drainage was performed, with evacuation of approximately 1 L of purulent material.
Microbiological assessment	Culture of the drained purulent material grew *Staphylococcus aureus*. Detailed antimicrobial susceptibility testing results and subsequent antibiotic adjustments were not available in the medical record.
One week after surgical drainage – Discharge	The postoperative course was uneventful, and the patient was discharged.
One-month follow-up – Recovery	The patient was asymptomatic, with complete clinical resolution of the infection.

## Discussion

Iliopsoas abscess is an uncommon clinical entity and represents an exceptionally rare complication following cesarean delivery, with only a limited number of postpartum cases reported in the literature [6,7,9,10]. Because of its deep anatomical location and nonspecific clinical presentation, diagnosis is frequently delayed, increasing the risk of extensive local spread and severe infectious complications [3].

Iliopsoas abscesses are generally classified as primary or secondary. Primary abscesses result from hematogenous dissemination and are most commonly caused by *Staphylococcus aureus*, whereas secondary abscesses arise from contiguous spread of infection from adjacent organs or may occur in association with postoperative infectious complications [1,2].

The clinical manifestations of iliopsoas abscess are often nonspecific. Although the classical triad of fever, back pain, and limp has been described, it is observed in only a minority of patients, contributing to delayed diagnosis [11]. Pain may radiate to the hip or thigh because of irritation of the lumbar plexus [12]. In our patient, persistent fever associated with severe lumbar pain, flank swelling, and spontaneous thigh fistulization strongly suggested an advanced infectious process requiring prompt investigation.

Contrast-enhanced CT remains the imaging modality of choice because it accurately defines the location, extent, and complexity of the abscess while identifying fistulous tracts and involvement of adjacent structures [3,13]. Although ultrasound may provide an initial assessment, its diagnostic performance is limited by bowel gas, while magnetic resonance imaging may be useful in selected circumstances [7]. In the present case, CT was essential for demonstrating communication between the retroperitoneal iliopsoas collection and the large lumbar subcutaneous abscess, thereby facilitating appropriate surgical planning.

Management of iliopsoas abscess requires prompt initiation of appropriate antimicrobial therapy combined with adequate drainage of the collection [14]. Image-guided percutaneous drainage may be appropriate in selected patients, whereas open surgical drainage may be required for large, complex, multiloculated, or fistulized collections, or when percutaneous drainage is not feasible or is unlikely to provide adequate source control [14,15]. In our patient, the extensive size of the abscess, its fistulization, and its communication with the retroperitoneal space supported the decision to perform surgical drainage. This approach, combined with antimicrobial therapy, was followed by rapid clinical improvement and complete recovery.

The precise pathogenesis of the iliopsoas abscess in our patient cannot be established with certainty. Primary iliopsoas abscess typically results from hematogenous dissemination and is frequently associated with *Staphylococcus aureus*, whereas secondary abscess results from contiguous spread from an adjacent infectious focus [1,2]. In the present case, the isolation of *Staphylococcus aureus* is compatible with hematogenous dissemination. However, the close temporal relationship with cesarean delivery, the history of prolonged rupture of membranes exceeding 24 hours, and the concomitant clinical and ultrasonographic findings suggestive of postpartum endometritis also raise the possibility of secondary extension from a pelvic infectious focus. The coexistence of features supporting both hematogenous and contiguous mechanisms therefore prevents definitive determination of the route of infection in this patient.

Prolonged rupture of membranes may have contributed indirectly by increasing the risk of ascending intrauterine infection and subsequent postpartum endometritis, potentially providing a source for deeper pelvic or retroperitoneal infectious spread. Nevertheless, a direct causal relationship between prolonged rupture of membranes and the development of the iliopsoas abscess cannot be established from this single observation.

Given the prolonged rupture of membranes and subsequent findings suggestive of postpartum endometritis, timely recognition and appropriate antimicrobial treatment of postpartum infection may theoretically reduce the risk of progression to deeper infectious complications. However, whether earlier or broader antimicrobial therapy following cesarean delivery would have prevented the development of the iliopsoas abscess in this patient remains uncertain and cannot be determined from a single case. Antimicrobial therapy should therefore be guided by established obstetric indications, microbiological findings when available, and the patient's clinical condition rather than by this isolated observation.

This case highlights the importance of considering iliopsoas abscess in postpartum women presenting with persistent fever associated with lumbar or flank pain, swelling, or lower-limb symptoms, particularly when the clinical course is atypical or fails to improve with initial treatment. In our patient, cross-sectional imaging, multidisciplinary management, appropriate antimicrobial therapy, and timely drainage were associated with a favorable clinical outcome. Although this observation illustrates the potential value of early recognition and adequate source control when postpartum iliopsoas abscess is suspected, conclusions regarding optimal diagnostic and therapeutic management should be interpreted cautiously given the single-case nature of this report.

## Conclusions

Iliopsoas abscess is an exceptionally rare but potentially serious postpartum complication following cesarean delivery. This case illustrates that iliopsoas abscess should be considered among the differential diagnoses in postpartum patients presenting with persistent fever associated with lumbar, flank, hip, or thigh symptoms, particularly when the clinical course is atypical for more common puerperal infections.

In this patient, contrast-enhanced CT was essential for identifying the iliopsoas involvement, defining the extensive lumbar and thigh extension, and demonstrating the fistulous communication, thereby guiding therapeutic management. The favorable outcome following antimicrobial therapy and surgical drainage illustrates the potential value of timely multidisciplinary management in extensive collections.

The temporal association with cesarean delivery following prolonged rupture of membranes and the presence of postpartum endometritis suggest a possible secondary infectious mechanism, although hematogenous dissemination cannot be excluded. Because this report describes a single patient, no causal relationship or broadly generalizable conclusions regarding risk factors, prevention, or optimal management can be drawn. Nevertheless, this case may increase clinical awareness of this uncommon diagnosis and of the importance of considering deep retroperitoneal infection in postpartum patients with persistent fever and atypical lumbar or lower-limb symptoms.
